# Opioid Receptors in Psychedelia: Indirect Serotonergic Modulation of Direct KOR Activation by Salvinorin A

**DOI:** 10.3390/biomedicines14020476

**Published:** 2026-02-21

**Authors:** Maximiliano Ganado, Carmen Rubio, Javier Pérez-Villavicencio, Norma Serrano, Héctor Romo-Parra, Ángel Lee, Moisés Rubio-Osornio

**Affiliations:** 1 Department of Neurophysiology, National Institute of Neurology and Neurosurgery, Mexico City 14269, Mexico; abraxasplnts@gmail.com (M.G.); mrubio@innn.edu.mx (C.R.); javierperezvillavicencio@gmail.com (J.P.-V.); nasmy_sr@yahoo.com.mx (N.S.); hector.romo.parra@gmail.com (H.R.-P.); 2 Basic Sciences and Engineering Division, Iztapalapa Campus, Department of Electrical Engineering, Metropolitan Autonomous University, Mexico City 09340, Mexico; 3 Santa Fe Campus, Ibero-American University, Mexico City 01376, Mexico; 4 National Institute of Public Health, Cuernavaca 62100, Mexico; dr_angel_lee@yahoo.de; 5 Department of Neurochemistry, National Institute of Neurology and Neurosurgery, Mexico City 14269, Mexico

**Keywords:** psychedelics, salvinorin A, kappa opioid receptor (KOR), neuropharmacology neuroplasticity

## Abstract

The neuropharmacology of psychedelics has traditionally focused on serotonergic mechanisms, particularly 5-HT2A receptor activation. However, this paradigm incompletely explains the diversity of neurobiological and therapeutic effects observed across psychedelic compounds. Non-classical psychedelics such as salvinorin A, the primary active constituent of *Salvia divinorum*, challenge this framework through direct kappa opioid receptor (KOR) agonism, representing a serotonin-independent pathway to altered consciousness. This review systematically examines the role of the endogenous opioid system in mediating psychedelic effects, with emphasis on salvinorin A’s unique KOR-dependent mechanisms. We synthesized preclinical and clinical evidence from in vitro studies, genetically modified animal models, optogenetic circuit dissection, and human neuroimaging trials. Salvinorin A’s selective KOR activation is characterized by pronounced β-arrestin-biased signaling, distinguishing it from endogenous dynorphins and classical KOR agonists. This produces rapid receptor desensitization, transient functional plasticity, and profound dissociative effects mediated through thalamocortical disruption, mesolimbic dopaminergic suppression, and fragmentation of large-scale brain networks. Classical serotonergic psychedelics indirectly engage opioid systems through downstream 5-HT2A signaling, contributing to analgesic and mood-regulatory effects via secondary MOR/DOR modulation. Despite being a potent opioid agonist, salvinorin A exhibits low abuse potential due to aversive phenomenology, dopaminergic suppression, and absence of positive reinforcement in animal models. Incorporating opioid receptor pharmacology into psychedelic neuroscience expands mechanistic understanding beyond serotonin-centric models, revealing multiple neurochemical pathways capable of inducing therapeutically relevant altered states. This framework enables rational development of biased KOR ligands and establishes salvinorin A as a paradigmatic model for non-serotonergic psychedelia with applications in treatment-resistant depression, addiction, and chronic pain.

## 1. Introduction

Throughout a significant portion of the 20th century, the scientific exploration of psychedelics was impeded by social, political, and legal obstacles. Over the past two decades, however, a renewed interest in these compounds has emerged, driven by accumulating evidence of their therapeutic potential in treatment-resistant psychiatric conditions, including major depressive disorder, post-traumatic stress disorder, anxiety associated with terminal illness, and substance use disorders [1,2,3]. This resurgence has coincided with significant progress in neuroscience, pharmacology, and neuroimaging, enabling a more precise characterization of the neural and molecular mechanisms underlying psychedelic effects [4,5,6].

Historically, the neuropharmacology of psychedelics has been interpreted predominantly through a serotonergic framework. The structural similarity between serotonin (5-hydroxytryptamine, 5-HT) and classical psychedelics such as psilocybin, lysergic acid diethylamide (LSD), N,N-dimethyltryptamine (DMT), psilocin, mescaline, and 5-MeO-DMT supported the hypothesis that their effects are primarily mediated by activation of 5-HT receptors, particularly the 5-HT2A subtype [7,8]. These compounds share key pharmacophoric features—an indole or indole-like aromatic core and an ethylamine side chain—that enable recognition by the 5-HT2A receptor, as depicted in Table 1, which provides complete chemical nomenclature and structural representations highlighting these pharmacophoric elements.

In divergence from these serotonergic compounds, the identification and characterization of non-classical psychedelics have highlighted the limitations of a serotonin-centered perspective. Salvinorin A, a potent and highly selective agonist of the kappa opioid receptor (KOR) [9,10,11], and ibogaine, a compound that interacts with multiple neurotransmitter systems [12,13], illustrate pharmacological profiles that diverge markedly from those of classical serotonergic psychedelics. These compounds underscore the need to expand existing models to incorporate additional neuromodulatory systems.

In this context, the endogenous opioid system is a critical modulatory network interfacing with serotonergic, dopaminergic, and affective processes altered during psychedelic states. This system plays a central role in the regulation of pain, reward, mood, stress responsivity, and motivational states, processes that are consistently altered during psychedelic experiences [14,15]. Understanding how opioid receptor signaling interfaces with broader neurotransmitter networks is therefore essential for developing a more integrative framework of psychedelic neurobiology.

This review advances a systems-level perspective in which classical and non-classical psychedelic compounds are compared explicitly through the lens of dynamic interactions between opioid, serotonergic, dopaminergic, and glutamatergic signaling pathways. This work highlights how differential modes of receptor engagement direct versus indirect, canonical versus biased shape circuit-level dynamics, transient versus sustained plasticity, and ultimately the qualitative structure of altered conscious experience. By integrating preclinical, molecular, and network-level evidence, this study establishes a unifying conceptual framework in which salvinorin A serves as a paradigmatic model for non-serotonergic psychedelia mediated by direct opioid receptor activation.

### 1.1. Structural Logic and Organization of This Review

This review adopts a layered analytical approach that progresses from molecular pharmacology to systems-level neurobiology. To facilitate conceptual integration, we introduce salvinorin A early in the exposition (Section 1.2) as a concrete pharmacological case that anchors subsequent discussion of opioid receptor organization and function. This reverse-translational structure reflects a deliberate pedagogical choice: salvinorin A represents the most selective and behaviorally potent example of direct KOR-mediated psychedelia, making it an ideal reference point for interpreting the broader role of the opioid system in altered states of consciousness.

Following this introduction, Section 2 provides an in-depth overview of the endogenous opioid system, including receptor subtypes, anatomical distribution, intracellular signaling pathways, and functional roles in pain, reward, aversion, and stress. Section 3 examines the functional convergence between classical serotonergic psychedelics and the opioid system, demonstrating how 5-HT2A receptor activation indirectly recruits opioid signaling to modulate perception, affect, and therapeutic outcomes. Finally, Section 4 examines salvinorin A in mechanistic detail, synthesizing multilevel evidence to delineate κ-opioid receptor-mediated psychedelia as a distinct neuropharmacological paradigm.

This organizational framework allows for a progressive increase in analytical resolution while maintaining conceptual clarity and avoiding premature technical complexity. By anchoring abstract systems-level concepts in a specific, well-characterized compound, we aim to facilitate both expert analysis and broader accessibility.

### 1.2. Salvinorin A: A Unique Non-Classical Psychedelic

Salvinorin A, the principal psychoactive compound isolated from *Salvia divinorum*, represents a distinctive paradigm within the neuropharmacology of non-classical psychedelics. In contrast to traditional hallucinogens, salvinorin A is a nitrogen-free diterpene [9,16], making it the first naturally occurring, highly potent opioid agonist that lacks a basic nitrogen atom in its chemical structure. This structural uniqueness contributes to its atypical pharmacological profile and has positioned it as a valuable tool for investigating opioid-mediated alterations of consciousness independently of serotonergic pathways.

The primary mechanism of action of salvinorin A is its highly selective agonism at the kappa opioid receptor (KOR), with binding affinities in the nanomolar range and negligible activity at mu (MOR) and delta (DOR) opioid receptors, as well as at classical serotonergic, dopaminergic, or glutamatergic receptors [9,10,11,17]. This exceptional selectivity enables the dissociation of KOR-specific effects from those mediated by other neuromodulatory systems, providing a pharmacological “clean slate” for studying pure kappa opioid contributions to perception, affect, and consciousness.

Beyond its receptor selectivity, salvinorin A exhibits pronounced biased signaling properties, preferentially engaging β-arrestin-mediated pathways over canonical G protein signaling [18,19,20]. This signaling bias distinguishes salvinorin A from both endogenous dynorphin peptides and classical synthetic KOR agonists, with profound implications for both the phenomenology and the temporal dynamics of its psychoactive effects.

At the experiential level, salvinorin A produces intense, short-lived alterations in consciousness characterized by profound dissociation, perceptual distortion, loss of self-reference, and disruption of spatial and temporal coherence. Unlike classical serotonergic psychedelics, which often evoke states of heightened insight, emotional openness, and mystical experience, salvinorin A typically induces dysphoric, disorienting, and non-euphoric states that users rarely describe as pleasurable [21]. This distinctive phenomenological profile reflects the compound’s unique receptor pharmacology and circuit-level effects, which are detailed extensively in Section 4.

The study of salvinorin A expands the conceptual framework of psychedelic research by demonstrating that profound alterations in perception, affect, and consciousness can arise through direct opioid receptor activation rather than serotonergic mechanisms. As such, salvinorin A serves as a critical comparative model alongside classical psychedelics, revealing mechanistic diversity within the broader psychedelic spectrum and offering insights into the neurobiology of dissociative and altered states of consciousness [11,22].

### 1.3. Scope and Objectives

This review synthesizes current knowledge on the role of the opioid system in psychedelic neuropharmacology, with particular emphasis on salvinorin A as a paradigmatic case of KOR-mediated psychedelia. Specific objectives include:

1. Characterizing the intracellular signaling pathways, receptor pharmacology, and neuronal effects that underline salvinorin A’s psychoactive properties, with emphasis on biased β-arrestin signaling and its functional consequences (Section 4.2).
2. Mapping the anatomical distribution and functional roles of KOR across thalamocortical, mesolimbic, and limbic circuits, and examining how salvinorin A disrupts large-scale brain networks to produce dissociative states (Section 2.2, Section 4.3 and Section 4.4).
3. Contrasting the direct KOR-mediated effects of salvinorin A with the indirect opioid recruitment observed in classical serotonergic psychedelics, highlighting mechanistic divergence and phenomenological implications (Section 3).
4. Evaluating the translational potential of KOR-targeted compounds for psychiatric and neurological disorders, addressing safety concerns, abuse liability, and the development of biased agonists with improved therapeutic profiles (Section 4.6 and Section 4.7).

By integrating molecular, circuit, and phenomenological perspectives, this work establishes a comprehensive framework for understanding how the opioid system acting either directly or indirectly shapes the neurobiology of psychedelic states and their therapeutic potential.

## 2. The Opioid System: Organization and Neurobiological Functions

This review introduces salvinorin A before discussing the opioid system to anchor neurobiological concepts within a concrete pharmacological case. Salvinorin A represents the most selective and behaviorally potent example of direct KOR-mediated psychedelia, making it an ideal reference point for interpreting opioid receptor organization, signaling properties, and circuit-level roles. This structure reflects a reverse-translational logic, moving from a specific compound-driven phenomenon toward a generalized systems-level understanding of opioid-mediated modulation of consciousness.

### 2.1. Opioid Receptors and Endogenous Ligands

The endogenous opioid system is a major neuromodulatory network of the central nervous system (CNS) that plays a fundamental role in the regulation of pain, emotion, motivation, stress responses, and neuronal homeostasis. This system comprises three classical opioid receptors MOR, DOR, and KOR all of which belong to the G protein–coupled receptor (GPCR) family and predominantly couple to Gi/o proteins [15].

Opioid receptors are activated by endogenous peptide ligands derived from distinct precursor proteins. Endorphins, generated from proopiomelanocortin (POMC), exhibit high affinity for MOR; enkephalins, derived from proenkephalin, preferentially activate MOR and DOR; and dynorphins, produced from prodynorphin, are the principal endogenous ligands for KOR. The relative selectivity between ligands and receptor subtypes provides substantial functional diversity, allowing fine-tuned regulation of neural circuits in physiological and pathological contexts [23,24].

At the molecular level, opioid receptor activation inhibits adenylate cyclase activity, leading to reduced intracellular cyclic AMP concentrations and decreased protein kinase A (PKA) signaling [15,25]. In parallel, opioid receptors modulate ion channel function by opening G protein–coupled inwardly rectifying potassium (GIRK) channels [26,27] and inhibiting voltage-gated calcium channels [28], resulting in reduced neuronal excitability and diminished presynaptic neurotransmitter release. These mechanisms constitute a shared cellular substrate of opioid signaling, with functional consequences that vary markedly depending on receptor subtype, cellular context, and circuit architecture [28,29].

### 2.2. Anatomical Organization and Distribution of Opioid Receptors

Opioid receptors display a highly specific and heterogeneous distribution throughout the CNS, which underlies the diversity of behavioral and physiological effects elicited by their activation. MOR expression is particularly prominent in regions involved in analgesia and reward processing, including the spinal cord, brainstem, thalamus, nucleus accumbens, and prefrontal cortex. DORs are widely distributed in cortical and limbic areas, including the hippocampus and amygdala, where they contribute to emotional regulation, stress resilience, and neuroprotective mechanisms [30,31].

KORs are abundantly expressed in the prefrontal cortex, thalamus, hippocampus, amygdala, striatum, and ventral tegmental area (VTA)—regions that mediate perception, affective processing, motivation, and stress responses central to salvinorin A’s phenomenology [32]. This distributed expression pattern positions KOR as a key modulator of affective states, stress responsivity, and conscious experience across multiple brain systems.

### 2.3. Functions of the Opioid System in Synaptic Modulation

Within the CNS, the opioid system primarily functions as a modulatory system rather than a fast synaptic transmitter. Activation of opioid receptors at presynaptic terminals suppresses the release of excitatory neurotransmitters such as glutamate and, in specific circuit contexts, inhibitory neurotransmitters including GABA. These effects are highly circuit-dependent and allow opioids to dynamically regulate information flow within neural networks [33,34].

Opioid receptors modulate synaptic gain, oscillatory activity, and neuronal synchronization by altering the excitation–inhibition balance. These actions are particularly significant in cortical networks, where opioid modulation can alter sensory processing, cognitive integration, and global brain states. Moreover, sustained or repeated opioid receptor activation can induce changes in synaptic plasticity, affecting mechanisms such as long-term potentiation (LTP) and long-term depression (LTD), thereby shaping experience-dependent network reorganization [35].

### 2.4. Opioid System, Reward, Aversion, and Stress

One of the most extensively characterized roles of the opioid system is its involvement in reward and aversion. MOR activation is closely associated with analgesia, pleasure, and positive reinforcement and mediates many of the euphoric effects of classical opioid drugs. In contrast, KOR activation is consistently linked to dysphoric, aversive, and stress-related states, functioning as a counter-regulatory mechanism to dopaminergic reward signaling [36].

Activation of KORs by endogenous dynorphins suppresses dopamine release in the nucleus accumbens and prefrontal cortex, a mechanism that is physiologically engaged during acute and chronic stress. This dynorphin–KOR axis plays a central role in emotional regulation, stress adaptation, and the modulation of motivational states, providing a neurobiological substrate for aversion and negative affect [14].

### 2.5. Intracellular Signaling and β-Arrestin Activation

In addition to canonical Gi/o protein-dependent signaling, opioid receptors engage β-arrestin-mediated intracellular pathways that regulate receptor desensitization, internalization, and downstream kinase activation. β-Arrestin signaling can activate pathways involving ERK1/2, p38 MAPK, and JNK, which are implicated in transcriptional regulation, neuronal plasticity, and long-term cellular adaptations [14,37].

Notably, β-arrestin-dependent KOR signaling has been strongly associated with dysphoric and stress-related effects, motivating the development of biased agonists that selectively favor beneficial signaling pathways while minimizing adverse outcomes. This conceptual framework is directly relevant to unconventional KOR agonists such as salvinorin A, whose signaling profile differs from that of both endogenous ligands and traditional opioid drugs [38].

### 2.6. Interactions of the Opioid System with Other Neurotransmitter Systems

The opioid system interacts extensively with dopaminergic, glutamatergic, GABAergic, and serotonergic neurotransmitter systems, enabling integrated modulation of neuronal activity across multiple levels of organization. Through these interactions, opioid receptor activation can exert complex effects on perception, affect, cognition, and conscious experience.

Opioid modulation of thalamocortical circuits regulates sensory integration and information flow to the cortex. By altering thalamic gating and cortical responsiveness, opioid signaling can induce states of sensory decoupling that are highly relevant for understanding non-classical psychedelic phenomena [39,40,41]. Therapeutic strategies targeting psychoactive compounds require selective engagement of specific receptor subtypes and intracellular signaling pathways to achieve precise neuromodulatory effects.

### 2.7. Relevance of the Opioid System in Non-Classical Psychedelic Effects

The functional organization of the opioid system, and particularly the kappa opioid receptor, provides a robust neurobiological framework for understanding how certain compounds can induce altered states of consciousness independently of direct serotonergic activation. Selective KOR engagement can profoundly influence neuronal excitability, synaptic transmission, sensory integration, large-scale brain network connectivity, and subjective experience [22,42,43].

Within this framework, the opioid system emerges not only as a regulator of pain and affective states, but also as a central modulator of conscious experience. This perspective establishes the conceptual foundation for analyzing non-classical psychedelics such as salvinorin A and for integrating opioid receptor signaling into broader models of psychedelic neurobiology.

## 3. Interaction Between Classical Psychedelics and the Opioid System

Classical psychedelics exhibit primary affinity for serotonergic receptors, particularly 5-HT2A. However, their neurobiological and behavioral effects cannot be explained exclusively by serotonergic signaling. A substantial body of preclinical and clinical research demonstrates that serotonergic receptor activation engages in functional interactions with other neuromodulatory systems, most notably the endogenous opioid system, which contributes significantly to the modulation of perception, affective states, and conscious experience [44,45,46,47,48].

Serotonergic signaling recruits downstream neuromodulatory mechanisms that shape both qualitative and quantitative features of the psychedelic state. The opioid system functions as a critical integrative component, modulating sensory processing, emotional valence, and network-level dynamics.

### 3.1. Functional Convergence Between the Serotonergic and Opioid Systems

At the cellular level, activation of the 5-HT2A receptor by classical psychedelics initiates intracellular signaling cascades involving phospholipase C activation, intracellular calcium mobilization, and engagement of kinases such as protein kinase C and ERK1/2. These signaling pathways converge with opioid receptor-mediated mechanisms within key neuronal populations, particularly cortical pyramidal neurons and GABAergic interneurons that play central roles in sensory integration and cognitive control [49].

Evidence demonstrates that serotonergic activation may modulate endogenous opioid systems through several mechanisms. Serotonergic and opioid signaling pathways converge at multiple circuit levels, with both systems showing reciprocal interactions in cortical and limbic regions [50,51]. Through these interactions, classical psychedelics may indirectly engage opioid receptor signaling, potentially resulting in secondary modulation of MOR and DOR pathways, and under specific neurophysiological conditions, recruitment of KOR signaling [52].

### 3.2. Preclinical Evidence: Opioid Modulation of Psychedelic Effects

Preclinical studies provide evidence supporting involvement of the opioid system in behavioral effects induced by classical psychedelics. Pharmacological studies have demonstrated that opioid receptor antagonists can modulate certain behavioral responses to serotonergic hallucinogens [53], and opioid system activity has been implicated in reinforcement and affective components of psychedelic experiences [54]. These findings suggest that opioid signaling may function as a downstream or parallel modulator of serotonergic activation, potentially contributing to specific aspects of the psychedelic phenotype [55].

Within this modulatory framework, MOR and DOR signaling have been primarily associated with positive affective, anxiolytic, and analgesic components of the psychedelic experience. In contrast, KOR recruitment appears to be involved in more complex and context-dependent effects, including modulation of emotional valence, stress responsiveness, and aspects of self-processing [56,57,58,59]. Differential engagement of opioid receptor subtypes likely contributes to heterogeneous subjective and behavioral outcomes of classical psychedelics.

### 3.3. Serotonergic–Opioid Interactions in Analgesic and Antidepressant Effects

This interaction is clearest in pain and mood modulation. Classical psychedelics such as psilocybin demonstrate analgesic and antidepressant-like effects in preclinical models and clinical studies, partially through indirect engagement of the endogenous opioid system [60,61].

The analgesic effects of psychedelics may involve interactions between serotonergic and opioid systems. Both 5-HT2A and 5-HT1A receptors are expressed in pain-processing regions including the prefrontal cortex, hippocampus, and periaqueductal gray, where they can modulate descending pain control pathways [62,63]. While direct evidence for psychedelic-induced endorphin release remains limited, clinical observations of reduced pain perception following psychedelic exposure [64] suggest potential engagement of endogenous analgesic mechanisms, possibly involving MOR and DOR pathways [63,65]. These interactions may contribute to reductions in both sensory and affective components of pain.

### 3.4. Salvia Divinorum as a Contrasting and Complementary Model

Salvinorin A, as the principal psychoactive constituent of *Salvia divinorum*, provides a contrasting yet complementary framework for examining the relationship between psychedelics and the opioid system. Unlike classical psychedelics, salvinorin A acts as a direct and highly selective agonist of the kappa opioid receptor, with minimal involvement of serotonergic receptors. This pharmacological specificity allows for a dissociation between effects arising from primary opioid receptor activation and those emerging from serotonergic–opioid interactions [9,66,67].

At the cellular level, direct KOR activation by salvinorin A produces robust inhibition of neuronal excitability through modulation of ion channels and suppression of excitatory neurotransmitter release. At the network level, these actions result in pronounced disorganization of thalamocortical and limbic circuits, generating states of perceptual dissociation and profound alterations in self-representation [21].

Comparison between classical psychedelics and salvinorin A highlights the dual role of the opioid system in altered states of consciousness: as a secondary modulator of serotonergic effects in classical compounds, and as a primary mediator of phenomenology in the case of *Salvia divinorum* [68,69].

### 3.5. Biased Signaling and Neuronal Plasticity

**Figure 1:**
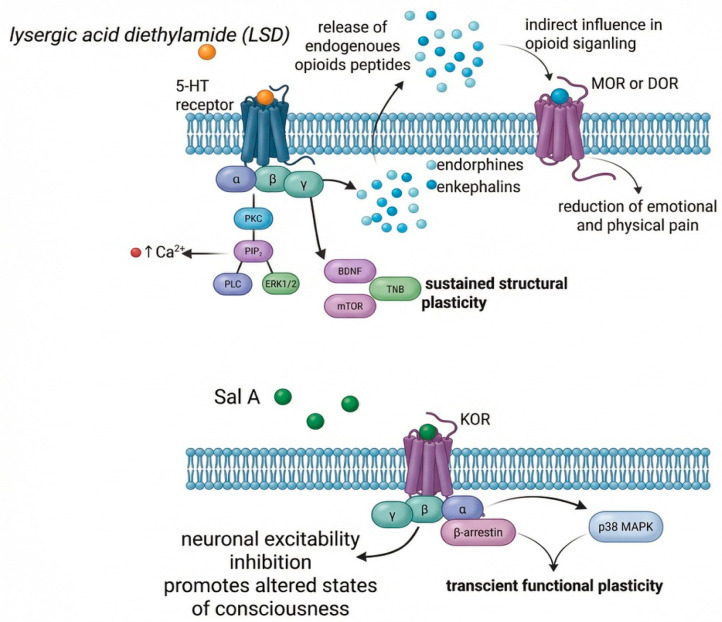
Contrasting mechanisms of neuronal plasticity: classical serotonergic psychedelics versus salvinorin A. (**Top**) Classical psychedelics (e.g., LSD) activate 5-HT receptors, triggering Gq/11 signaling (PLC, PKC, ERK1/2, Ca^2+^ mobilization) and releasing endogenous opioids that indirectly engage MOR/DOR. This activates BDNF–TrkB–mTOR pathways, driving sustained structural plasticity (dendritic growth, synaptogenesis) and reducing emotional/physical pain. (**Bottom**) Salvinorin A (Sal A) directly activates KOR through Gi/o-coupled, β-arrestin-biased signaling, activating p38 MAPK and producing transient functional plasticity (rapid synaptic changes without structural remodeling). KOR activation inhibits neuronal excitability and induces dissociative altered states. This comparison illustrates how receptor engagement and signaling bias determine plasticity duration and therapeutic potential. Abbreviations: BDNF, brain-derived neurotrophic factor; Ca^2+^, calcium; DOR, δ-opioid receptor; ERK1/2, extracellular signal-regulated kinase 1/2; 5-HT, 5-hydroxytryptamine; KOR, κ-opioid receptor; LSD, lysergic acid diethylamide; MAPK, mitogen-activated protein kinase; MOR, μ-opioid receptor; mTOR, mechanistic target of rapamycin; PKC, protein kinase C; PLC, phospholipase C; Sal A, salvinorin A; TrkB, tropomyosin receptor kinase B.

Both classical psychedelics and salvinorin A induce changes in neuronal plasticity, although through distinct intracellular mechanisms. Serotonergic psychedelics promote sustained structural plasticity mediated by BDNF, mTOR, and TrkB signaling pathways, whereas KOR activation by salvinorin A induces transient functional plasticity through β-arrestin-dependent pathways [14,70,71,72]. The divergent signaling cascades underlying these contrasting forms of plasticity—including the release of endogenous opioid peptides by classical psychedelics and the direct β-arrestin-biased KOR activation by salvinorin A—are exhibited in Figure 1, with detailed molecular mechanisms elaborated in Section 4.2.

These functional distinctions indicate that temporal dynamics and qualitative features of therapeutic effects depend on convergence or divergence of serotonergic and opioid signaling in regulating synaptic and network plasticity. Importantly, signaling bias alone does not determine therapeutic outcome; the regional distribution and circuit-level organization of receptor expression critically shape both phenomenology and clinical potential.

### 3.6. Implications for the Neurobiology of Consciousness

The interaction between serotonergic and opioid systems expands the conceptual framework of the neurobiology of consciousness, demonstrating that altered states do not arise from engagement of a single neurotransmitter system [73,74]. Within this expanded model, *Salvia divinorum* and salvinorin A serve as valuable neuropharmacological tools for elucidating how kappa opioid signaling alone can generate profoundly transformative conscious experiences [22].

Classical and non-classical psychedelics converge on modulation of large-scale brain connectivity and sensory integration through distinct neurochemical pathways. This functional convergence underscores the central role of the opioid system, acting either directly or indirectly, in the full expression of psychedelic effects.

The mechanistic divergence between serotonin-dominant and opioid-dominant pathways is further developed in Section 4, where salvinorin A is examined in detail as the paradigmatic case of direct KOR-mediated psychedelia, alongside other non-classical compounds such as ibogaine.

## 4. Non-Classical Psychedelics and Direct Activation of Opioid Receptors

Non-classical psychedelics comprise a heterogeneous group of compounds capable of inducing altered states of consciousness through neuropharmacological mechanisms that do not rely primarily on serotonergic receptor activation. Within this category, salvinorin A, the principal active compound of *Salvia divinorum*, represents the most clearly defined and best-characterized example of a psychedelic whose effects are mediated directly through the opioid system, specifically via activation of the kappa opioid receptor (KOR) [9,11]. These compounds demonstrate that serotonin is not the sole neuromodulatory system capable of profoundly altering perception, cognition, and conscious experience.

### 4.1. Salvinorin A as a Paradigm of Direct Activation of the Opioid System

The exceptional KOR selectivity of salvinorin A makes it a unique tool for studying pure kappa opioid signaling effects on perception, affect, and consciousness. From a pharmacokinetic perspective, the high lipophilicity of salvinorin A enables rapid penetration into the central nervous system [75,76], resulting in an almost immediate onset of action and a short-lived yet extremely intense psychoactive profile [21,77]. Rapid KOR desensitization and receptor internalization explain this distinctive temporal pattern [78].

### 4.2. Cellular and Molecular Mechanisms of KOR Activation by Salvinorin A

#### 4.2.1. Gi/o Protein-Dependent Signaling and Neuronal Excitability

At the cellular level, activation of the KOR by salvinorin A, a Gi/o protein–coupled receptor, leads to inhibition of adenylate cyclase, resulting in reduced intracellular cAMP levels and decreased protein kinase A (PKA) activity [14,15]. These molecular events directly influence neuronal excitability by promoting membrane hyperpolarization through activation of G protein–coupled inwardly rectifying potassium (GIRK) channels and inhibition of N-type and P/Q-type voltage-gated calcium channels [26,27,28]. As a result, presynaptic release of excitatory neurotransmitters, particularly glutamate, is attenuated.

These cellular mechanisms effectively reduce synaptic gain across cortical and subcortical circuits, profoundly disrupting sensory integration and cognitive processing through widespread neuronal hyperpolarization and diminished glutamatergic transmission [26,28].

#### 4.2.2. β-Arrestin Biased Signaling

The psychedelic and dissociative effects of salvinorin A cannot be attributed only to fast G protein-mediated actions. Sustained activation of β-arrestin-2-dependent pathways plays a critical role, leading to the phosphorylation of kinases such as p38 MAPK, JNK, and ERK1/2, as well as modulation of plasticity-related factors including BDNF and Arc [18,19,25]. These signaling cascades are linked to transcriptional regulation, synaptic remodeling, and short- to medium-term forms of neural plasticity.

The pronounced β-arrestin bias of salvinorin A results from ligand-specific receptor conformations that favor GRK recruitment and sustained β-arrestin-2 engagement [10,18,19,79,80]. This signaling preference distinguishes salvinorin A from both endogenous dynorphin peptides and classical synthetic KOR agonists. The β-arrestin-mediated cascades promote rapid receptor desensitization and internalization, limit prolonged G protein-mediated signaling, and mediate cellular stress responses [20,81]. These mechanisms provide a cellular explanation for the intense yet short-lived psychoactive effects of salvinorin A, as well as for its distinctive dissociative and aversive phenomenology [82,83].

Although extensive transcriptomic analyses specifically for salvinorin A remain limited, studies of KOR signaling suggest the involvement of immediate early genes and activity-dependent factors in patterns distinct from those induced by serotonergic psychedelics [84,85]. Unlike psilocybin or LSD, which robustly promote structural plasticity through BDNF–TrkB and mTOR signaling pathways, salvinorin A induces transient, context-dependent functional plasticity defined by the swift reconfiguration of excitatory and inhibitory synaptic transmission without sustained dendritic growth or long-term synaptogenesis [70,86].

**Figure 2:**
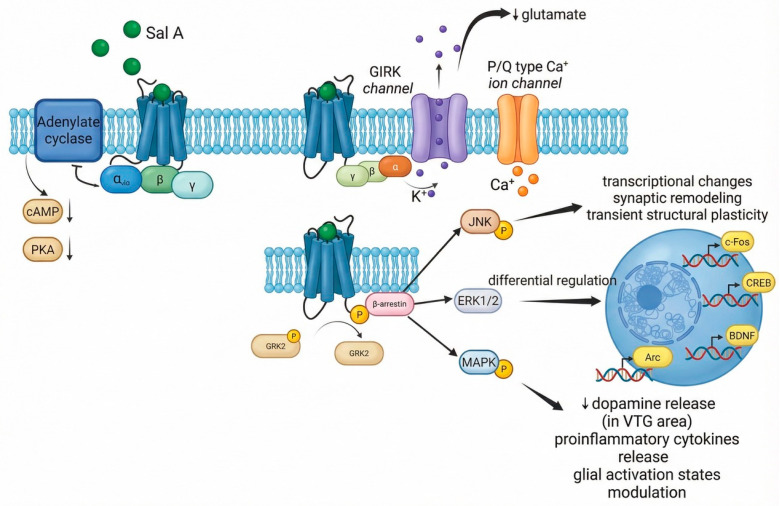
**Intracellular signaling pathways activated by salvinorin A through κ-opioid receptor engagement.** Salvinorin A (Sal A) activates the κ-opioid receptor (KOR) to engage two parallel signaling cascades. (**Left**) Canonical Gi/o protein-dependent signaling: KOR activation inhibits adenylate cyclase, reducing intracellular cAMP levels and decreasing protein kinase A (PKA) activity, resulting in broad inhibitory effects on cellular excitability. (**Right**) Biased β-arrestin-dependent signaling: Salvinorin A preferentially recruits β-arrestin-2, which promotes G protein-coupled receptor kinase 2 (GRK2)-mediated receptor phosphorylation and internalization. β-arrestin engagement activates mitogen-activated protein kinase (MAPK) cascades including JNK, ERK1/2, and p38 MAPK, leading to phosphorylation and activation of downstream effectors. (**Top**) Membrane effects: KOR activation opens G protein-coupled inwardly rectifying potassium (GIRK) channels and inhibits P/Q-type voltage-gated calcium (Ca^2+^) channels, producing neuronal hyperpolarization and reduced presynaptic glutamate release. (**Bottom right**) Nuclear and cellular consequences: Convergence of MAPK pathways on transcription factors (c-Fos, CREB) and plasticity-related genes (BDNF, Arc) drives transcriptional changes, synaptic remodeling, and transient functional plasticity. Additional effects include suppression of dopamine release in the ventral tegmental area (VTA), modulation of proinflammatory cytokine release, and regulation of glial activation states. This pronounced β-arrestin bias distinguishes salvinorin A from endogenous dynorphin peptides and classical synthetic KOR agonists, providing a molecular basis for its intense yet short-lived psychoactive effects and distinctive dissociative phenomenology. Abbreviations: Arc, activity-regulated cytoskeleton-associated protein; BDNF, brain-derived neurotrophic factor; cAMP, cyclic adenosine monophosphate; CREB, cAMP response element-binding protein; ERK1/2, extracellular signal-regulated kinase 1/2; GIRK, G protein-coupled inwardly rectifying potassium; GRK2, G protein-coupled receptor kinase 2; JNK, c-Jun N-terminal kinase; KOR, κ-opioid receptor; MAPK, mitogen-activated protein kinase; PKA, protein kinase A; Sal A, salvinorin A; VTA, ventral tegmental area. 2; JNK, c-Jun N-terminal kinase; MAPK, mitogen-activated protein kinase; PKA, protein kinase A; Sal A, salvinorin A; VTA, ventral tegmental area.

The parallel engagement of canonical Gi/o protein-dependent and biased β-arrestin-mediated signaling pathways by salvinorin A, along with their convergent and divergent downstream effects, is illustrated schematically in Figure 2. This figure shows the molecular basis for salvinorin A’s distinctive pharmacological profile, including rapid receptor desensitization, transient functional plasticity, and the integration of neuronal, glial, and circuit-level effects that collectively generate its characteristic dissociative phenomenology.

### 4.3. Synaptic, Glial, and Neuronal Plasticity Effects

#### 4.3.1. Transient Functional Plasticity at Synaptic and Network Levels

The transient functional plasticity induced by salvinorin A manifests at multiple organizational levels. At the synaptic level, sustained KOR activation alters the excitation–inhibition balance through modulation of both glutamatergic and GABAergic transmission. At the network level, these changes produce temporary reorganization of thalamocortical and limbic connectivity, generating marked alterations in perception and self-processing [70,71]. This plasticity is temporally constrained, closely coupled to receptor kinetics, and rapidly reversible following drug clearance—a mechanistic basis for the brief yet profoundly disruptive conscious states associated with KOR-dominant psychedelia.

#### 4.3.2. Glial Modulation and Neuroimmune Interactions

KOR signaling extends beyond neurons to glial populations, particularly microglia and astrocytes [87,88]. Activation of KOR in microglial cells modulates the release of proinflammatory cytokines and regulates glial activation states, suggesting a role for kappa opioid signaling in neuroimmune modulation [88,89,90]. These observations motivate development of selective or biased KOR agonists for chronic pain, inflammation, and stress-related disorders that dissociate therapeutic benefits from salvinorin A’s extreme psychoactive effects [91].

#### 4.3.3. Evidence from Genetically Modified Animal Models

Definitive evidence for the KOR-selective mechanism of salvinorin A comes from studies using genetically modified mice. In animals with targeted deletion of the *Oprk1* gene encoding the κ-opioid receptor (KOR knockout mice), salvinorin A fails to elicit its characteristic behavioral effects. Across doses typically used in behavioral assays (0.3–3.0 mg/kg, i.p.), KOR knockout mice show a complete absence of key salvinorin A–induced responses observed in wild-type controls.

Specifically, wild-type mice exhibit dose-dependent locomotor suppression with peak effects occurring 5–10 min post-injection, whereas KOR knockout mice display no such suppression, demonstrating KOR-dependent motor effects [9,11]. Within comparable dose ranges, salvinorin A produces robust conditioned place aversion in wild-type mice—an effect completely abolished in KOR knockout animals—confirming that its aversive properties are mediated exclusively via KOR signaling [14,36]. Similarly, in tail-flick and hot-plate assays, salvinorin A induces antinociceptive responses in wild-type but not KOR knockout mice; these effects are reversed by the selective KOR antagonist nor-binaltorphimine (nor-BNI), but not by MOR or DOR antagonists [11,17].

Importantly, KOR knockout mice retain normal responses to MOR agonists (morphine) and 5-HT2A agonists (DOI, psilocybin), demonstrating that the absence of salvinorin A effects is due to specific loss of KOR rather than generalized deficits in drug responsiveness [9,17].

In conditional knockout studies where KOR expression is selectively deleted in dopaminergic neurons of the ventral tegmental area (VTA), salvinorin A’s ability to suppress dopamine release in the nucleus accumbens is significantly attenuated, while effects on other neurotransmitter systems remain intact. This dissociation demonstrates that dopaminergic suppression is a direct consequence of KOR activation on dopamine neurons rather than a secondary effect of altered cortical or limbic activity [39,92].

#### 4.3.4. Circuit-Specific Mechanisms Revealed by Optogenetics

Recent optogenetic and chemogenetic approaches have enabled a precise dissection of κ-opioid receptor (KOR) function within defined neural circuits, revealing circuit-specific contributions to the behavioral and cognitive effects of salvinorin A.

In the ventral tegmental area (VTA), selective optogenetic manipulation of KOR-expressing dopaminergic neurons—using Cre-dependent strategies in DAT-Cre or TH-Cre mouse lines—has demonstrated a causal role for this population in mediating aversive and motivational outcomes. Specifically, optogenetic activation of these neurons recapitulates key behavioral effects of salvinorin A, including conditioned place aversion and diminished motivation for natural rewards, whereas optogenetic inhibition or chemogenetic silencing via designer receptors exclusively activated by designer drugs (DREADDs) effectively abolishes these aversive responses, confirming their necessity in salvinorin A–induced behavioral suppression [39,93].

Beyond the mesolimbic system, chemogenetic inhibition of KOR-expressing neurons in the medial prefrontal cortex significantly attenuates salvinorin A–induced impairments in cognitive flexibility and working memory. These findings indicate that cortical KOR signaling contributes directly to the dissociative and cognitive effects of salvinorin A, operating at least in part independently of dopaminergic suppression in subcortical reward circuits [21,94].

Complementing these observations, KOR expression within thalamic relay nuclei—particularly the mediodorsal and ventrobasal nuclei—has been implicated in the modulation of sensory processing and gating. Localized microinfusion of salvinorin A into these thalamic regions reproduces core features of sensory decoupling observed following systemic administration, whereas focal infusion of KOR antagonists into the thalamus selectively blocks the sensory effects of systemically administered salvinorin A [95].

These circuit-level studies demonstrate that the complex phenomenology of salvinorin A does not arise from KOR activation within a single anatomical locus, but rather from coordinated and spatially distributed receptor engagement across mesolimbic, cortical, and thalamic networks.

### 4.4. Alterations of Neural Circuits and Networks of Consciousness

#### 4.4.1. Interaction with the Dopaminergic System and the Dynorphin–KOR Axis

A central aspect of salvinorin A’s neuropharmacology is its interaction with the mesocorticolimbic dopaminergic system. Activation of KORs in neurons of the ventral tegmental area (VTA) suppresses dopamine release in the nucleus accumbens and prefrontal cortex, an effect mediated through the endogenous dynorphin–KOR axis. In vivo microdialysis studies in freely moving rats demonstrate that systemic administration of salvinorin A produces rapid and dose-dependent reductions in extracellular dopamine concentrations in the nucleus accumbens, with peak suppression of 40–60% occurring 10–20 min post-injection [39,92,96]. This effect is completely reversed by pretreatment with the selective KOR antagonist nor-binaltorphimine, confirming KOR-mediated mechanisms.

Experimental evidence indicates that salvinorin A induces swift and region-specific neurochemical alterations across multiple neurotransmitter systems. In the nucleus accumbens, dopamine levels are markedly reduced, with reported decreases of approximately 40–60%, emerging within 5–10 min of administration and persisting for 30–60 min [39,96]. In parallel, glutamatergic transmission in the prefrontal cortex is significantly attenuated, showing a 25–40% reduction with a rapid onset of less than 5 min and a duration of approximately 20–40 min [26,28,97]. Serotonergic signaling in the prefrontal cortex appears to be less consistently affected; several studies report only a modest increase, on the order of 15–25%, which is thought to arise indirectly through GABAergic disinhibition rather than direct receptor-mediated mechanisms [98]. In accordance with this interpretation, GABA release exhibits region-dependent modulation, with decreased inhibitory output from ventral tegmental area interneurons contributing to the suppression of dopaminergic neuron activity, while parallel effects have been observed in the amygdala [39].

Critically, these neurotransmitter changes follow a hierarchical temporal sequence: KOR activation produces immediate hyperpolarization of dopamine neurons (within 1–2 min), followed by decreased dopamine release (5–10 min), with secondary effects on glutamate and serotonin emerging downstream (10–30 min). This temporal ordering supports a model in which direct KOR-mediated dopamine suppression constitutes the primary event, with subsequent alterations in other neurotransmitter systems representing circuit-level consequences rather than parallel independent effects [39,92,96].

This reduction in dopaminergic signaling has been linked to dysphoria, anhedonia, and altered motivational processing, distinguishing the subjective profile of salvinorin A from that of serotonergic psychedelics, which tend to enhance dopaminergic tone indirectly [14,39,92]. This negative modulation of reward circuitry contributes to the loss of agency, self-dissolution, and non-euphoric quality characteristic of salvinorin A–induced states [21,39,92].

Taken together, preclinical findings indicate that the neurobiological effects of salvinorin A arise from a hierarchical sequence of events rather than from parallel, independent neurotransmitter changes. Direct activation of KOR constitutes the primary molecular event, leading to rapid suppression of dopaminergic firing in mesolimbic and mesocortical pathways via Gi/o-mediated inhibition and dynorphin–KOR signaling. Secondary modulation of serotonergic and glutamatergic transmission appears to emerge downstream of this initial dopaminergic suppression, altering salience attribution, sensory integration, and affective valence. This causal ordering distinguishes salvinorin A from classical serotonergic psychedelics, in which 5-HT2A receptor activation initiates cortical excitation and indirectly recruits opioid signaling. Such mechanistic divergence provides a neurochemical basis for the distinct phenomenological profiles associated with KOR-dominant versus serotonin-dominant psychedelic states [99,100].

#### 4.4.2. Thalamocortical Circuits and Sensory Integration

At the circuit level, KOR activation by salvinorin A exerts profound effects on thalamocortical pathways responsible for sensory integration and the construction of conscious experience. KOR signaling reduces excitatory transmission from the thalamus to the cortex, producing sensory decoupling that manifests as perceptual distortions, alterations in spatial and temporal perception, and dissociative phenomena [9,13].

Functional neuroimaging studies indicate that salvinorin A induces a transient disorganization of large-scale brain networks, with connectivity patterns distinct from those observed under serotonergic psychedelics. Notably, marked disruption of the default mode network is accompanied by fragmentation of communication between frontoparietal and limbic regions [21,94]. These system-level effects converge on thalamocortical circuitry, resulting in sensory decoupling and destabilization of higher-order cortical integration.

**Figure 3:**
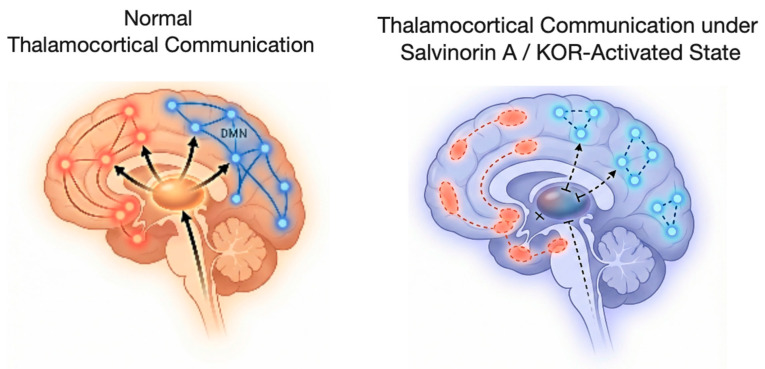
**Thalamocortical decoupling and high-order network reorganization induced by Salvinorin A.** Comparative schematic of functional thalamocortical communication under basal conditions and during kappa opioid receptor(KOR) activation. (**Left**) In the normal state, the thalamus functions as a central integrative hub, supporting ascending sensory transmission and coherent connectivity across the default mode network (DMN, blue), as well as limbic and frontoparietal networks (red). (**Right**) Direct activation of KOR by Salvinorin A inhibits ascending sensory pathways (dashed lines), producing thalamic gating that leads to fragmentation of the DMN and dissociation of higher-order cortical networks. This system-level reorganization provides a mechanistic framework for understanding dissociative states and the transient disruption of maladaptive connectivity patterns associated with treatment-resistant depression, addiction, and chronic pain.

At the systems level, salvinorin A produces profound alterations in thalamocortical circuitry, leading to a marked reduction in the transmission of sensory information to the cerebral cortex. This sensory decoupling is associated with extreme perceptual distortions, dissociative experiences, and a pronounced disruption of self-referential processing. Functional neuroimaging studies indicate that direct KOR activation results in fragmentation of large-scale brain networks, including the default mode network (DMN), with connectivity patterns that differ substantially from those induced by serotonergic psychedelics [4,75]. Multiple mechanistically distinct neurobiological pathways converge on altered states of consciousness depending on the primary neuromodulatory system engaged. This network-level reorganization induced by salvinorin A is presented in Figure 3.

### 4.5. Ibogaine: An Atypical Psychedelic with Complex Pharmacology

Ibogaine, an indole alkaloid extracted from the root bark of Tabernanthe iboga, represents another important paradigm of a non-classical psychedelic. Unlike the pharmacological specificity of salvinorin A, ibogaine is characterized by notable receptor promiscuity, interacting simultaneously with multiple neurotransmitter systems [12,13,101,102].

At the molecular level, ibogaine acts as a non-competitive antagonist of NMDA receptors, particularly at the MK-801 binding site [101]. This property confers dissociative effects similar to ketamine, although with a distinctive phenomenological profile characterized by prolonged oneiric states and profound emotional processing. Additionally, ibogaine exhibits significant affinity for several serotonergic receptor subtypes (including 5-HT2A), opioid receptors (especially KOR and MOR), monoamine transporters, and nicotinic cholinergic receptors α3β4 [12,102].

This complex pharmacology produces dissociative, introspective, oneiric, and anti-addictive effects. Crucially, ibogaine’s principal metabolite, noribogaine (12-hydroxyibogamine), possesses a considerably longer half-life (approximately 24–48 h) and contributes substantially to long-term therapeutic effects, particularly in the treatment of substance use disorders [103,104].

The mechanism by which ibogaine interrupts opioid dependence involves multiple levels of action: (1) Dual activity on KOR and NMDA blockade in the ventral tegmental area and nucleus accumbens may restore dopaminergic balance altered by chronic opioid use [102,103]; (2) Ibogaine induces structural reorganization of cortico-limbic circuits associated with reward and stress processing [105]; and (3) Ibogaine dramatically reduces opioid withdrawal symptoms, facilitating the transition to abstinence without the severe distress characteristic of conventional detoxification [103,104].

Despite its therapeutic potential, ibogaine presents significant safety challenges. The most critical is its cardiotoxicity, manifested primarily as QT interval prolongation and risk of potentially lethal ventricular arrhythmias, particularly torsades de pointes [106,107]. This risk is amplified in individuals with preexisting cardiac disease, electrolyte imbalances, or concomitant use of other QT-prolonging medications. These safety concerns have motivated the development of synthetic analogs with more favorable pharmacological profiles. Notably, recently developed oxa-iboga alkaloids retain anti-addictive activity while showing significantly reduced cardiotoxicity in animal models [108].

#### Preliminary Clinical Evidence and Contrast with Salvinorin A

Observational studies in uncontrolled clinical settings have reported sustained abstinence rates of 30–50% at 12 months in individuals treated with ibogaine for opioid dependence, figures notably superior to conventional treatments [103,109]. However, the absence of randomized controlled clinical trials, combined with safety concerns, limits the interpretation of these findings and widespread clinical implementation.

The comparison between ibogaine and salvinorin A illustrates two distinct neuropharmacological strategies for inducing altered states of consciousness. Salvinorin A exerts highly selective effects via KOR, whereas ibogaine engages multiple neurotransmitter systems; salvinorin A produces brief effects (5–30 min), while ibogaine induces prolonged states (12–24 h, with residual effects lasting days); salvinorin A is associated with intense dissociation and depersonalization, whereas ibogaine facilitates introspective states and autobiographical emotional processing; and salvinorin A lacks known cardiac toxicity, in contrast to the significant cardiovascular risks associated with ibogaine. These differences underscore the need for conceptual frameworks that accommodate mechanistic diversity within the psychedelic spectrum, as systematically summarized in Table 2.

### 4.6. Abuse Potential, Addiction, and the Role of β-Arrestin Signaling

#### 4.6.1. The Paradox of Low Abuse Potential in an Opioid Agonist

The involvement of β-arrestin signaling in salvinorin A-mediated KOR activation raises important questions regarding addiction liability and receptor regulation. In classical opioid pharmacology, β-arrestin recruitment is often associated with receptor desensitization, internalization, and the development of tolerance, processes that can contribute to compulsive drug use [28,112]. However, extrapolating this framework to salvinorin A requires caution. In the context of KOR signaling, β-arrestin-dependent pathways are strongly linked to dysphoric and aversive states, suppression of dopaminergic reward signaling, and negative reinforcement rather than euphoria. Thus, while β-arrestin engagement by salvinorin A may accelerate receptor desensitization, it simultaneously promotes subjective experiences that actively discourage repeated use, thereby decoupling receptor internalization from addiction risk [127].

Salvinorin A presents a pharmacological paradox regarding abuse potential. Traditionally, opioid receptor agonists, particularly MOR, exhibit high addictive potential due to activation of mesolimbic reward circuits. However, despite being a potent opioid agonist, salvinorin A shows a fundamentally different abuse profile in epidemiological and laboratory studies [22].

Patterns of Salvia divinorum use differ markedly from substances with high addictive potential. Population surveys indicate that repeated use is infrequent, with most users experiencing the substance once or a few times [21,116]; patterns of compulsive seeking or escalated use characteristic of addiction are not observed; the subjective experience is frequently described as intense, challenging, and not inherently pleasurable, which contrasts with the positive reinforcement associated with classical addictive substances. Several mechanisms explain this resistance to addiction development.

#### 4.6.2. Dopaminergic Suppression and Aversion

As discussed in previous sections, KOR activation in the ventral tegmental area suppresses dopamine release in the nucleus accumbens and prefrontal cortex [14,39,92]. This effect is opposite to the dopaminergic increase that mediates the positive reinforcement of addictive drugs such as MOR-selective opioids, psychostimulants, and alcohol. The resulting subjective experience frequently includes dysphoria, restlessness, and aversive affective states that limit motivation for repeated use.

Animal model studies consistently demonstrate that KOR agonists do not sustain self-administration and, in fact, produce conditioned aversion in place preference paradigms [14,36]. In intravenous self-administration studies, rats and non-human primates with established histories of opioid self-administration fail to acquire or maintain salvinorin A self-administration across a wide range of doses (0.01–1.0 mg/kg/infusion), a pattern distinct from the robust self-administration observed with MOR agonists at comparable doses [122]. In contrast to morphine or fentanyl, which produce increasing response rates with dose escalation, salvinorin A produces active avoidance behavior, with animals developing strategies to minimize drug exposure.

In conditioned place preference/aversion paradigms, salvinorin A consistently produces dose-dependent place aversion at doses that produce KOR activation (0.3–3.0 mg/kg in mice), with aversion scores inversely proportional to dose. This contrasts sharply with the place preference induced by MOR agonists and the mixed preference/aversion patterns of DOR agonists [14,36,96].

#### 4.6.3. β-Arrestin Signaling: Risk Factor or Protection?

Salvinorin A’s biased signaling toward β-arrestin raises a complex question regarding addictive potential. Activation of β-arrestin-2 by GPCR agonists, including KOR, leads to phosphorylation of the receptor by G protein-coupled receptor kinases (GRKs), reducing response to agonist [37,38]; clathrin-mediated endocytosis that removes receptors from the cell surface [78]; and degradation of internalized receptors, reducing the density of available receptors [78].

These processes could generate rapid tolerance, potentially motivating increased dose or frequency of use a pattern associated with dependence development. However, β-arrestin signaling in the KOR context appears to protect against addiction through several mechanisms [58].

First, β-arrestin signaling of KOR has been specifically associated with dysphoric and aversive effects [33,71], which constitute a powerful deterrent for repeated use. Secondly, the relationship between β-arrestin and addiction has been studied primarily in the MOR context. MOR agonists biased toward β-arrestin show complex effects: while they may reduce adverse effects such as respiratory depression, β-arrestin’s contribution to MOR’s addictive potential remains controversial [81]. Extrapolating these findings to KOR, which mediates opposite effects in reward circuits, is problematic. Third, the combination of ultrarapid onset, high potency, and brief duration may limit the exposure window necessary to induce sustained neuroadaptive changes associated with dependence; and fourth, unlike MOR-selective opioids, physiological withdrawal syndromes have not been reported after cessation of salvinorin A or Salvia divinorum use, even in users with histories of repeated use [120,121,128].

Although theoretical concerns have been raised regarding β-arrestin-mediated κ-opioid KOR desensitization, available empirical data indicate that the desensitization kinetics of salvinorin A act to constrain, rather than enhance, its abuse liability. Repeated administration at short inter-dose intervals (approximately 15–30 min) produces a rapid and pronounced acute tolerance, such that second and third exposures result in a 60–80% attenuation of both behavioral and neurochemical effects. Notably, this form of acute tolerance develops substantially faster than that observed with morphine or other MOR agonists [78,129].

In parallel, receptor-level studies demonstrate a rapid recovery of KOR responsiveness following salvinorin A exposure, with functional signaling restored within 4–6 h across most brain regions. This recovery contrasts sharply with the prolonged desensitization and downregulation observed after sustained morphine administration, which can require 24–72 h for functional recovery. Such kinetics are consistent with efficient receptor recycling rather than receptor degradation or long-term loss of signaling capacity [78].

Importantly, repeated daily administration of salvinorin A over extended periods (7–14 days) fails to induce behavioral sensitization, a phenomenon characterized by the progressive amplification of drug effects and commonly associated with psychostimulants and certain psychedelics. Instead, behavioral responses to salvinorin A remain stable or exhibit mild attenuation, indicating an absence of maladaptive neuroadaptations that would otherwise promote escalation of use or compulsive drug-seeking behavior [11,129].

#### 4.6.4. Biased KOR Agonists: Therapeutic Perspectives

Development of KOR agonists with biased signaling (G protein-biased, minimizing β-arrestin) has emerged as a strategy to retain potential therapeutic effects (analgesia, anti-addiction) while reducing dysphoric adverse effects [81]. Experimental compounds such as bivalent triazoles and salvinorin B derivatives have demonstrated sustained analgesia in neuropathic and visceral pain models; marked reduction in dysphoric and aversive effects compared to unbiased agonists; and absence of incapacitating sedation. These advances suggest that precise modulation of downstream signaling pathways of KOR can dissociate therapeutic from adverse effects.

#### 4.6.5. Considerations on KOR in Dopaminergic Neurons

KOR is expressed in dopaminergic neurons of the VTA [14,39]. Theoretically, KOR desensitization in these neurons could result in rebound dopaminergic disinhibition after cessation of salvinorin A exposure, potentially creating a neurochemical state favorable for substance seeking.

Nonetheless, empirical evidence does not support this scenario: Microdialysis studies in rodents have not demonstrated rebound increases in dopamine in nucleus accumbens after cessation of KOR agonists; and the rapid kinetics of salvinorin A (t½ < 10 min in primates [75]) may be insufficient to induce lasting neuroadaptive changes in dopaminergic systems.

On balance, available evidence suggests that salvinorin A presents low abuse potential despite being a potent opioid agonist. This distinctive profile results from the convergence of multiple factors: selectivity for KOR (dopaminergic suppression vs. activation); predominantly aversive subjective experience; absence of positive reinforcement in animal models; epidemiological patterns of non-compulsive use; and lack of withdrawal syndrome [128].

Biased signaling toward β-arrestin likely contributes to the aversive effects that limit repeated use, rather than promoting dependence development. So, additional research is necessary to fully characterize the long-term effects of repeated salvinorin A exposure, particularly in vulnerable populations or in contexts of co-use with other substances.

### 4.7. Conceptual and Therapeutic Implications

The study of non-classical psychedelics that directly engage opioid receptors has fundamentally reshaped contemporary models of psychedelia, demonstrating that the opioid system—particularly KOR signaling—can independently and profoundly modulate perception, emotion, and the sense of self. In this context, salvinorin A stands as a central neuropharmacological model for investigating non-serotonergic routes to altered states of consciousness [11,22].

These findings expand the conceptual framework of psychedelic neuroscience and have motivated development of biased opioid ligands capable of harnessing the therapeutic potential of KOR signaling while minimizing adverse effects. Such strategies may ultimately enable novel clinical applications grounded in opioid-mediated modulation of consciousness and affective processing [81,108].

The capacity of salvinorin A to rapidly modulate KOR signaling, neurotransmitter dynamics, synaptic plasticity, and large-scale brain networks challenges serotonin-centric models of psychedelia and expands the conceptual framework of consciousness neuroscience [66,73,130]. By demonstrating that profound alterations in perception, affect, and consciousness can arise independently of serotonergic mechanisms, salvinorin A establishes the opioid system—and particularly the kappa opioid receptor as a critical modulator of conscious experience [131]. This perspective provides a robust neurobiological foundation for analyzing non-classical psychedelics and for integrating opioid receptor signaling into broader models of psychedelic neurobiology.

## 5. Therapeutic Implications and Future Perspectives

The mechanistic analyses presented in Section 3 and Section 4 establish two complementary pathways through which opioid signaling modulates altered states of consciousness: indirect engagement via serotonergic activation (classical psychedelics) and direct KOR agonism (salvinorin A). These pathways converge on shared therapeutic targets—synaptic plasticity, affective regulation, network reorganization—while producing distinct phenomenological profiles and clinical implications [22,61,70,71,111,123].

Classical psychedelics induce sustained structural plasticity and positive affective states through 5-HT2A-mediated recruitment of MOR/DOR pathways (Section 3), whereas salvinorin A produces transient functional plasticity and dissociative states through direct β-arrestin-biased KOR signaling (Section 4). This duality positions the serotonergic–opioid interface as a central axis for therapeutic innovation, enabling strategies that target specific receptor combinations and signaling pathways for personalized psychiatric interventions [113].

Together, these findings position the serotonergic–opioid interface as a central axis for therapeutic innovation, spanning mood disorders, chronic pain, addiction, and trauma-related conditions. Current clinical evidence, mechanistic hypotheses, and translational progress in psychedelic–opioid modulation across major psychiatric and neurological indications are summarized in Table 3.

### 5.1. Implications for the Treatment of Chronic Pain

Chronic pain represents a major therapeutic challenge due to its multifactorial nature, encompassing sensory, emotional, and cognitive dimensions. Although modulation of the opioid system has long been central to pain management, the clinical utility of classical MOR agonists is limited by tolerance, dependence, and risk of misuse [132,137].

Psychedelic-opioid system interactions provide a novel approach to pain management [133,138]. Indirect engagement of opioid circuits by serotonergic psychedelics may enable modulation of affective and cognitive components of pain without the liabilities associated with chronic opioid administration [64,139]. Additionally, the study of salvinorin A has inspired the development of biased KOR agonists that aim to retain analgesic and anti-stress properties while minimizing dysphoric and psychotomimetic effects [20,81]. These approaches show promise for refractory pain syndromes and pain associated with inflammation or chronic stress.

### 5.2. Treatment-Resistant Depression and Mood Disorders

Treatment-resistant depression is associated with profound disturbances in synaptic plasticity, large-scale network organization, and emotional regulation [22,114]. Classical psychedelics such as psilocybin have demonstrated the capacity to promote functional reorganization of brain networks, particularly through modulation of the default mode network (DMN) and related associative circuits [4,22,117].

Converging evidence implicates the opioid system—especially the dynorphin–KOR axis—in the pathophysiology of depression, notably in dimensions such as anhedonia and dysphoria [14,118,119]. Insights gained from studying salvinorin A–induced KOR activation have directly informed the development of KOR antagonists and partial modulators as candidate antidepressant therapies [124,125,140]. Paradoxically, investigation of the potent psychedelic KOR agonist salvinorin A has contributed to therapeutic strategies aimed at selectively inhibiting or fine-tuning this signaling pathway [124,125].

### 5.3. Addictions and Substance Use Disorders

Substance use disorders represent another domain in which the interaction between psychedelics and the opioid system is particularly salient. Opioid signaling plays a central role in reward, aversion, and stress-related circuitry, and its dysregulation contributes to craving, relapse, and vulnerability to stress [14,141].

Polypharmacological compounds such as ibogaine (discussed in Section 4.5) have demonstrated efficacy in reducing withdrawal symptoms and interrupting addictive behaviors, likely through simultaneous modulation of opioid, serotonergic, and dopaminergic systems [103,104,109]. However, concerns regarding cardiotoxicity have limited their widespread clinical adoption.

In contrast, classical psychedelics may support addiction treatment by indirectly modulating opioid circuits while promoting enduring changes in cognition, emotional processing, and learning [70,115]. Clinical studies of psilocybin-assisted therapy for alcohol use disorder have reported significant reductions indicated substantial decreases in heavy drinking days, and long-term follow-up studies in smoking cessation have shown sustained abstinence [134,135]. When integrated with structured psychotherapeutic frameworks, psychedelic-assisted interventions Psychedelic-assisted therapies, when combined with established psychotherapy frameworks, appear particularly well suited to addressing especially effective in targeting the behavioral and cognitive dimensions of addiction.

Recent systematic reviews have reinforced underscored the therapeutic potential of psychedelic assisted therapy across multiple substances of abuse, including alcohol, tobacco, and opioids [126,136,142], highlighting the importance of combining emphasizing the significance of integrating neuropharmacological modulation with psychological and contextual factors.

## 6. Conclusions

This review establishes the endogenous opioid system as a fundamental pillar of psychedelic neuropharmacology, transcending the conventional serotonin-centric paradigm. Non-classical psychedelics exemplified by salvinorin A demonstrate that direct KOR agonism constitutes a serotonin-independent pathway to altered consciousness, characterized by profound thalamocortical sensory decoupling and dissociative phenomenology distinct from the ego-dissolution induced by classical serotonergic compounds.

Classical psychedelics, while primarily acting through 5-HT2A receptors, indirectly recruit opioid pathways to modulate analgesia, affective valence, and network-level plasticity. This functional convergence between serotonergic and opioid systems—mediated through distinct molecular mechanisms and signaling biases—underlies therapeutic efficacy observed across treatment-resistant depression, substance use disorders, and chronic pain. Critically, opioid modulation enables transient functional plasticity without the prolonged structural synaptogenesis characteristic of serotonergic mechanisms, offering complementary temporal dynamics for psychiatric intervention.

This dual framework positions the dynorphin-KOR axis as a pivotal regulator of consciousness and motivational states, with profound implications for rational development of biased ligands that harness opioid signaling’s therapeutic potential while minimizing dysphoric and psychotomimetic liabilities. By integrating direct and indirect opioid engagement strategies, this expanded model provides a mechanistic foundation for next-generation psychedelic-based therapeutics tailored to specific neuropsychiatric endophenotypes.

## 7. Limitations and Future Directions

Despite compelling preclinical convergence, the field faces substantial translational limitations. Current evidence relies predominantly on rodent models and in vitro assays, which limit direct extrapolation to human phenomenology due to species-specific differences in opioid receptor distribution, signaling bias (G protein versus β-arrestin pathways), and large-scale circuit dynamics. Human data remain comparatively sparse, constrained by ethical considerations, small cohort sizes in neuroimaging studies (e.g., fMRI investigations of salvinorin A), and pronounced inter-individual variability in subjective responses shaped by set and setting, as well as genetic polymorphisms in opioid receptor genes.

Additional challenges arise from pharmacological confounders inherent to non-classical compounds. The polypharmacological profiles of agents such as ibogaine—exhibiting affinity for MOR, serotonin transporters, and ion channels—complicate causal attribution across molecular and circuit-level effects. Safety considerations further limit clinical translation, including the extreme intensity and brief duration of salvinorin A–induced states, ibogaine-associated cardiotoxicity, and uncertainties surrounding glial KOR involvement in neuroinflammatory modulation, underscoring the early stage of clinical validation for these approaches.

Methodological limitations include the absence of large-scale, double-blind randomized controlled trials capable of disentangling serotonergic–opioid interactions, as well as the lack of longitudinal studies tracking biomarkers of plasticity such as BDNF and Arc. These gaps constrain mechanistic inference and impede robust assessment of durability and clinical relevance.

Future research should prioritize multimodal human neuroimaging approaches including fMRI, EEG, and MEG to characterize real-time circuit dynamics during combined serotonergic–opioid states. Parallel pharmacodynamic studies of peripherally restricted or signaling-biased κ-opioid receptor agonists will be essential to dissociate therapeutic anti-stress and analgesic effects from centrally mediated psychotomimetic outcomes. Phase II and III clinical trials integrating psychedelic-assisted psychotherapy are warranted for opioid use disorder, mood disorders, and neuroinflammatory conditions, with particular emphasis on biomarker-guided patient stratification (e.g., dynorphin signaling, large-scale network connectivity metrics).

Finally, comparative pharmacoepidemiological analyses across classical and non-classical psychedelic compounds, coupled with computational modeling of biased signaling and epigenetic investigations of long-term plasticity, will be critical for refining this integrative framework and may ultimately enable paradigm-shifting therapeutic strategies in psychiatry and related fields.

## Figures and Tables

**Table 1:** Chemical nomenclature and structures of serotonin and classical serotonergic psychedelics.

Chemical Structure	CAS Number	Molecular Formula	Full Chemical Name (IUPAC)	Common Name
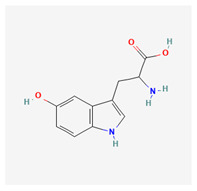	50-67-9	C_10_H_12_N_2_O	5-hydroxytryptamine; 3-(2 aminoethyl)-1H-indol-5-ol	5-HT (Serotonin)
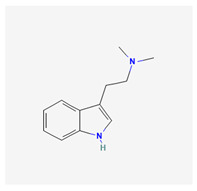	61-50-7	C_12_H_16_N_2_	N,N-dimethyltryptamine; 2-[2-(dimethylamino)ethyl]-1H-indole	DMT
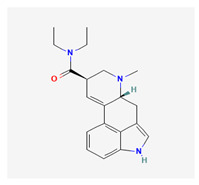	50-37-3	C_20_H_25_N_3_O	Lysergic acid diethylamide; (6aR,9R)-N,N-diethyl-7-methyl-4,6,6a,7,8,9-hexahydroindolo[4,3-fg]quinoline-9-carboxamide	LSD
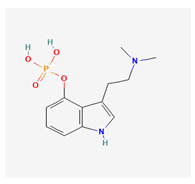	520-52-5	C_12_H_17_N_2_O_4_P	[3-[2-(dimethylamino)ethyl]-1H-indol-4-yl] dihydrogen phosphate	Psilocybin
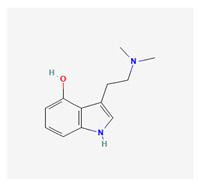	520-53-6	C_12_H_16_N_2_O	3-[2-(dimethylamino)ethyl]-1H-indol-4-ol	Psilocin
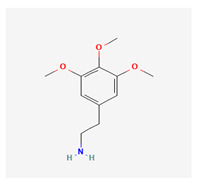	1954/4/6	C_11_H_17_NO_3_	2-(3,4,5-trimethoxyphenyl)ethanamine	Mescaline
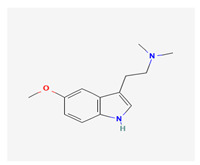	1019-45-0	C_13_H_18_N_2_O	5-methoxy-N,N-dimethyltryptamine; 2-[2-(dimethylamino)ethyl]-5-methoxy-1H-indole	5-MeO-DMT

All classical psychedelics share structural similarity with serotonin (5-hydroxytryptamine, 5-HT), featuring common pharmacophoric elements that enable 5-HT2A receptor recognition. Tryptamines (DMT, psilocybin, psilocin, 5-MeO-DMT) contain the canonical indole-ethylamine structure with variations in N-substitution and hydroxylation patterns. LSD features an ergoline scaffold a tetracyclic indole-related structure conferring high receptor affinity and prolonged duration of action. Mescaline presents a simplified phenylethylamine core, representing the minimal pharmacophore for 5-HT2A activation. The indole or indole-like aromatic core (highlighted in blue in structures) and ethylamine side chain (highlighted in green) constitute the essential structural motifs for serotonergic psychedelic activity. In contrast, salvinorin A, a diterpene from *Salvia divinorum*, lacks nitrogen atoms and serotonergic pharmacophores, acting instead through selective κ-opioid receptor (KOR) agonism, thereby demonstrating that non-serotonergic pathways can also mediate profound alterations in consciousness. 5-HT, 5-hydroxytryptamine (serotonin); 5-MeO-DMT, 5-methoxy-N,N-dimethyltryptamine; CAS, Chemical Abstracts Service; DMT, N,N-dimethyltryptamine; IUPAC, International Union of Pure and Applied Chemistry; LSD, lysergic acid diethylamide.

**Table 2:** Comparative neuropharmacology of classical and non-classical psychedelics: molecular mechanisms and circuit-level organization.

Key References	Ibogaine	Salvinorin A (*Salvia divinorum*)	Classical Psychedelics (Psilocybin, LSD, DMT)	Feature/Circuit
				MOLECULAR MECHANISMS
[94,99,101,105,110,111]	NMDA, KOR, MOR, 5-HT2A (multi-target)	κ-opioid receptor (KOR)	Ergoline5-HT2A (serotonin receptor)	Primary receptor target(s)
[101,105,111,112]	Mixed Gi/o, NMDA antagonism	Gi/o → adenylate cyclase inhibition	Gq/11 → PLC → IP3/DAG → Ca^2+^	Signal transduction
[112,113]	Variable/non-classical	β-arrestin-biased (strong recruitment)	G-protein predominant	Biased signaling
[94,96,97,98,105]	Multisystemic modulation	↓ Dopamine (mesolimbic), ↓ glutamate, indirect 5-HT modulation	↑ Glutamate, ↑ cortical excitation, indirect opioid release	Neurotransmitter modulation
				PLASTICITY
[111,113,114,115]	Context-dependent, mixed	Functional, transient (β-arrestin-dependent)	Structural, sustained (BDNF–TrkB–mTOR)	Type of plasticity
[99,101,105,116]	12–48 h (metabolite-dependent)	5–30 min	4–12 h	Duration of effects
				CIRCUIT-LEVEL ORGANIZATION
[94,97,105,110]	Indirect modulation via NMDA, serotonergic systems	Moderate KOR; executive disruption, dysphoria	High 5-HT2A expression; cognitive enhancement, self-processing modulation	Prefrontal–cortical networks
[94,116,117]	Variable, NMDA-dependent gating disruption	Strong KOR engagement; sensory decoupling, thalamic gating	Altered sensory integration, DMN modulation	Cortico-thalamic circuitry
[100,105,118,119]	Strong involvement; emotional memory reprocessing	Prominent KOR; stress and aversion processing	Emotional and contextual modulation, fear extinction	Limbic structures (amygdala, hippocampus)
[96,97,98,120]	Complex: KOR suppression + NMDA modulation	Direct suppression via VTA KOR activation	Indirect dopaminergic modulation (5-HT2A → DA)	Mesolimbic dopamine system
[92,98,118,119,121]	Marked involvement (anti-stress, anti-craving)	Primary engagement (dynorphin–KOR axis)	Secondary involvement	Stress/aversion networks
				CLINICAL PROFILE
[110,116]	Oneiric, introspective, autobiographical	Dissociative, dysphoric, depersonalizing	Mystical, insightful, ego-dissolution	Phenomenology
[92,106,107,122]	Low (aversive + toxic risk)	Very low (aversive)	Low	Abuse potential
[103,104,123,124,125,126]	Opioid/stimulant detoxification (observational)	KOR antagonists for depression; biased agonists for pain	Depression, anxiety, PTSD, addiction	Therapeutic applications
[106,107,108,116]	Cardiotoxicity (QT prolongation), neurotoxicity concerns	Intense but brief; no known organ toxicity	Generally favorable (psychological risks)	Safety profile

Integrative synthesis of molecular mechanisms, circuit-level organization, and clinical profiles of classical serotonergic psychedelics and non-classical compounds acting primarily through opioid receptor systems. Classical psychedelics engage 5-HT2A receptors to induce sustained structural plasticity and positive affective states, whereas salvinorin A produces transient functional plasticity and dysphoric dissociation through direct β-arrestin-biased KOR activation. Ibogaine represents a polypharmacological agent with complex multi-target engagement. Circuit-level effects reflect receptor distribution patterns and signaling properties, determining phenomenological and therapeutic profiles. Abbreviations: 5-HT2A, 5-hydroxytryptamine 2A receptor; BDNF, brain-derived neurotrophic factor; Ca^2+^, calcium; DAG, diacylglycerol; DMN, default mode network; IP3, inositol trisphosphate; KOR, κ-opioid receptor; MOR, μ-opioid receptor; mTOR, mechanistic target of rapamycin; NMDA, N-methyl-D-aspartate; PLC, phospholipase C; PTSD, post-traumatic stress disorder; TrkB, tropomyosin receptor kinase B; VTA, ventral tegmental area.

**Table 3:** Therapeutic applications of psychedelic-opioid modulation: current evidence and mechanisms.

Key References	Clinical Status	Proposed Mechanism	Salvinorin A/KOR Modulators	Classical Psychedelics (Indirect Opioid)	Condition
[61,123,124,125]	Clinical trials ongoing	DMN reorganization, BDNF ↑, dynorphin-KOR modulation	KOR antagonists: Phase 2 trials (e.g., FAST-MAS)	Psilocybin: Phase 2/3 trials, promising efficacy	Treatment-resistant depression
[64,81,132,133]	Preclinical/Early clinical	5-HT2A + MOR/DOR analgesia vs. direct KOR modulation	Biased KOR agonists: preclinical analgesia without dysphoria	LSD microdosing: preliminary evidence	Chronic pain
[103,134,135,136]	Clinical trials + observational	Network plasticity, motivational restructuring, withdrawal suppression	Ibogaine: opioid detoxification (observational)	Psilocybin: alcohol, tobacco cessation (Phase 2)	Substance use disorders
[3,61]	Clinical use (limited)	5-HT2A + opioid anxiolysis, existential reframing	Not established	Psilocybin: approved in some regions	Anxiety (terminal illness)
[60]	Near approval (MDMA)	Fear extinction, emotional processing	Not established	MDMA: Phase 3 trials (mixed 5-HT + opioid)	PTSD

Summary of therapeutic applications leveraging psychedelic-opioid system interactions. Classical psychedelics engage opioid systems indirectly through 5-HT2A activation, promoting sustained structural plasticity and positive affective states suitable for depression, addiction, and end-of-life anxiety. Direct KOR modulation (antagonism or biased agonism) offers complementary approaches for anhedonia-dominant depression and chronic pain with distinct temporal dynamics and phenomenological profiles. Abbreviations: BDNF, brain-derived neurotrophic factor; DMN, default mode network; KOR, κ-opioid receptor; MDMA, 3,4-methylenedioxymethamphetamine; PTSD, post-traumatic stress disorder.

## Data Availability

No new data were created or analyzed in this study.

## Abbreviations

5-HT	5-hydroxytryptamine (serotonin)
5-HT1A	5-hydroxytryptamine 1A receptor
5-HT2A	5-hydroxytryptamine 2A receptor
5-HT2C	5-hydroxytryptamine 2C receptor
Arc	activity-regulated cytoskeleton-associated protein
BDNF	brain-derived neurotrophic factor
c-Fos	cellular proto-oncogene fos
cAMP	cyclic adenosine monophosphate
CNS	central nervous system
CREB	cAMP response element-binding protein
D2	dopamine D2 receptor
DA	dopamine
DMN	default mode network
DMT	N,N-dimethyltryptamine
DOI	2,5-dimethoxy-4-iodoamphetamine
DOR	δ-opioid receptor
DREADDs	designer receptors exclusively activated by designer drugs
EEG	electroencephalography
ERK1/2	extracellular signal-regulated kinase 1/2
FAST-MAS	Fast-fail Trial in Mood and Anxiety Spectrum Disorders
fMRI	functional magnetic resonance imaging
GABA	gamma-aminobutyric acid
GIRK	G protein-coupled inwardly rectifying potassium (channels)
GPCR	G protein-coupled receptor
GRK	G protein-coupled receptor kinase
HIV-1	human immunodeficiency virus type 1
i.p.	intraperitoneal
JNK	c-Jun N-terminal kinase
KOR	κ-opioid receptor
LSD	lysergic acid diethylamide
LTD	long-term depression
LTP	long-term potentiation
MAPK	mitogen-activated protein kinase
MDA	3,4-methylenedioxyamphetamine
MDMA	3,4-methylenedioxymethamphetamine
MEG	magnetoencephalography
MK-801	dizocilpine, antagonist of NMDA receptor
MOR	μ-opioid receptor
mTOR	mechanistic target of rapamycin
NAc	nucleus accumbens
NMDA	N-methyl-D-aspartate (receptor)
nor-BNI	nor-binaltorphimine
p38 MAPK	p38 mitogen-activated protein kinase
PFC	prefrontal cortex
PKA	protein kinase A
PKC	protein kinase C
POMC	proopiomelanocortin
QT interval	time from the start of the Q wave to the end of the T wave (electrocardiogram)
RCT	randomized controlled trial
SERT	serotonin transporter
TrkB	tropomyosin receptor kinase B
VTA	ventral tegmental area
